# Land use influences macroinvertebrate community composition in boreal headwaters through altered stream conditions

**DOI:** 10.1007/s13280-016-0837-y

**Published:** 2016-11-01

**Authors:** Micael Jonsson, Ryan M. Burrows, Johan Lidman, Emma Fältström, Hjalmar Laudon, Ryan A. Sponseller

**Affiliations:** 1 Department of Ecology and Environmental Science, Umeå University, 901 87 Umeå, Sweden; 2 Department of Forest Ecology and Management, Swedish University of Agricultural Sciences, 901 83 Umeå, Sweden; 3 Australian Rivers Institute, Griffith University, Brisbane, QLD 4111 Australia; 4 Sweden Water Research AB, Ideon Science Park, Scheelevägen 15, 223 70 Lund, Sweden

**Keywords:** Aquatic insects, Biodiversity, Forestry, Functional traits

## Abstract

Land use is known to alter the nature of land–water interactions, but the potential effects of widespread forest management on headwaters in boreal regions remain poorly understood. We evaluated the importance of catchment land use, land cover, and local stream variables for macroinvertebrate community and functional trait diversity in 18 boreal headwater streams. Variation in macroinvertebrate metrics was often best explained by in-stream variables, primarily water chemistry (e.g. pH). However, variation in stream variables was, in turn, significantly associated with catchment-scale forestry land use. More specifically, streams running through catchments that were dominated by young (11–50 years) forests had higher pH, greater organic matter standing stock, higher abundance of aquatic moss, and the highest macroinvertebrate diversity, compared to streams running through recently clear-cut and old forests. This indicates that catchment-scale forest management can modify in-stream habitat conditions with effects on stream macroinvertebrate communities and that characteristics of younger forests may promote conditions that benefit headwater biodiversity.

**Electronic supplementary material:**

The online version of this article (doi:10.1007/s13280-016-0837-y) contains supplementary material, which is available to authorized users.

## Introduction

Headwater streams often account for the majority of network length, making them an important lotic habitat (Clarke et al. 2008). These small streams represent the primary interface between terrestrial and aquatic environments (Lowe and Likens 2005) and support key ecosystem processes, such as litter decomposition (Bilby and Likens 1980; Wallace et al. 1997) and nutrient retention (Bernhardt et al. 2005), that are crucial for the functioning of downstream lentic and lotic systems (Meyer and Wallace 2001). Further, headwater streams may house diverse species assemblages that are not only functionally important but also contribute to local and regional biodiversity (Finn et al. 2011). However, changed environmental conditions may lead to the loss of headwater species, altered community composition (Lowe and Likens 2005), and homogenization of communities resulting in reduced regional biodiversity (Meyer et al. 2007), with potential consequences for the functioning of these habitats (Vaughn 2010).

In boreal Sweden, headwater streams (draining catchments <1500 ha) represent more than 90 % of the total drainage length, yet remain poorly represented in nationwide monitoring and assessment programs (Bishop et al. 2008). Due to strong seasonal climate variability, these streams tend to be vulnerable to drought, bottom freezing, and floods (Malmqvist et al. 1999; Hoffsten 2003), requiring species to be adapted to highly dynamic hydrological conditions. Additionally, northern boreal headwaters are typically humic and naturally acidic (Laudon and Buffam 2008), nutrient poor (Bergström et al. 2008), and often shaded by dense, coniferous riparian vegetation (Naiman et al. 1987). In turn, these conditions regulate organic and inorganic resource availability and quality to macroinvertebrate consumers, through the input of relatively low-quality litter (Naiman et al. 1987), light and nutrient limitation of autotrophic production (Kiffney et al. 2004), and nutrient limitation of microbes (Burrows et al. 2015). Boreal headwater streams therefore represent rather unique combinations of harsh and limiting environmental conditions that likely constrain the productivity and richness of benthic communities (Annala et al. 2014).

Previous research aimed at understanding the factors controlling macroinvertebrate community composition in boreal streams has found combinations of several environmental and habitat variables to be important. For example, latitude, longitude, pH, and stream characteristics such as water velocity, width, and depth are often important determinants of macroinvertebrate community structure (Heino et al. 2003, 2014; Schmera et al. 2013). Moreover, variation in substrate composition (Heino et al. 2014) and concentrations of nutrients and dissolved organic carbon (DOC) (Göthe et al. 2014) may also drive patterns in benthic community composition.

Several studies from temperate regions show that external factors, such as riparian canopy openness, abundance of deciduous streamside vegetation, and catchment-scale land use affect stream habitats and communities (Allan 2004). In this context, research on boreal headwaters is underrepresented (but see Schmera et al. 2013; Heino et al. 2014). Given the strong reliance of headwater stream macroinvertebrates on terrestrial resources (Vannote et al. 1980; Webster and Benfield 1986; Richardson and Danehy 2007), any alterations to the terrestrial environment that result in quantitative or qualitative changes in allochthonous organic matter (OM) input, or levels of in-stream primary production (e.g. via increased canopy openness and/or nutrient inputs), may affect macroinvertebrate communities.

In addition to affecting the richness of stream assemblages, catchment properties also shape the functioning of these communities through effects on the diversity of species traits represented locally. Indeed, it is increasingly clear that the analysis of species traits adds additional insight to our understanding of how stream communities respond to environmental pressures and change (e.g. Poff et al. 2006). Knowing which functional traits are present in a community (and their relative abundance), and how the relative abundance of traits may change due to external influences, leads to a better understanding, and thus predictive ability, of how ecosystem functioning might be altered following changed environmental conditions (Poff 1997; Bonada et al. 2007). To enable predictions of how changed community composition affects ecosystem functioning, it is important to unravel drivers of those traits that are directly linked to the maintenance of ecosystem processes (e.g. filter feeders—filtration rate). Several previous studies have shown that ecosystem process rates and, hence, functioning can be related to species diversity (Vaughn 2010). However, functional traits are often shared among sets of species, and the occurrence of specific traits in a community may remain unchanged despite species losses or gains, due to functional redundancy among species (Rosenfeld 2002). Therefore, functional trait diversity is likely a more robust measure, compared to species richness, for understanding and predicting impacts of community change on ecosystem functioning (Poff 1997; Bonada et al. 2007).

In the Scandinavian boreal zone, land-use pressures on streams occur primarily through forest management, and in particular through clear-cutting (Laudon et al. 2011a), which increases the short-term concentrations of nutrients and DOC (Schelker et al. 2012, 2016), potentially elevates sediment loads (Futter et al. 2016), and reduces canopy cover and changes community composition of riparian vegetation (McKie and Malmqvist 2009). All these changes are known to influence stream macroinvertebrate structure and function (Zhang et al. 2009; Hoover et al. 2011; Schmera et al. 2013; Göthe et al. 2014; Heino et al. 2014). Effects of clear-cutting may be transient (Hoover et al. 2011) and/or difficult to detect (McKie and Malmqvist 2009), and likely change as adjacent managed forests regenerate and stream macroinvertebrate communities recover towards a pre-disturbance state (Stone and Wallace 1998; Liljaniemi et al. 2002). However, such long-term patterns in recovery may not be detected unless later stages of forest regeneration also are considered. Hence, studies that encompass all the stages of regeneration of managed boreal forests are required to detect the cumulative impact of forestry and assess how influential this type of land use is, compared to other factors, at shaping boreal headwater environments and macroinvertebrate communities (Zhang et al. 2009).

Here we ask whether the impacts on benthic invertebrate communities caused by boreal forest management are detectable when considered in conjunction with natural variation in land cover (e.g. percentage of lakes and mires in catchment), geographical variables (e.g. altitude, catchment size), and in-stream environmental conditions. To do this, we used 18 boreal headwater catchments in northern Sweden to investigate the influence of land use, land cover, and in-stream environmental conditions, in addition to influences of geographical variables, on stream macroinvertebrate community composition, and functional trait diversity. With this design, our aim was to investigate how gradients in catchment-scale land use and land-cover characteristics influence stream environmental conditions and, subsequently, macroinvertebrate communities.

## Materials and methods

### Study sites

**Fig. 1:**
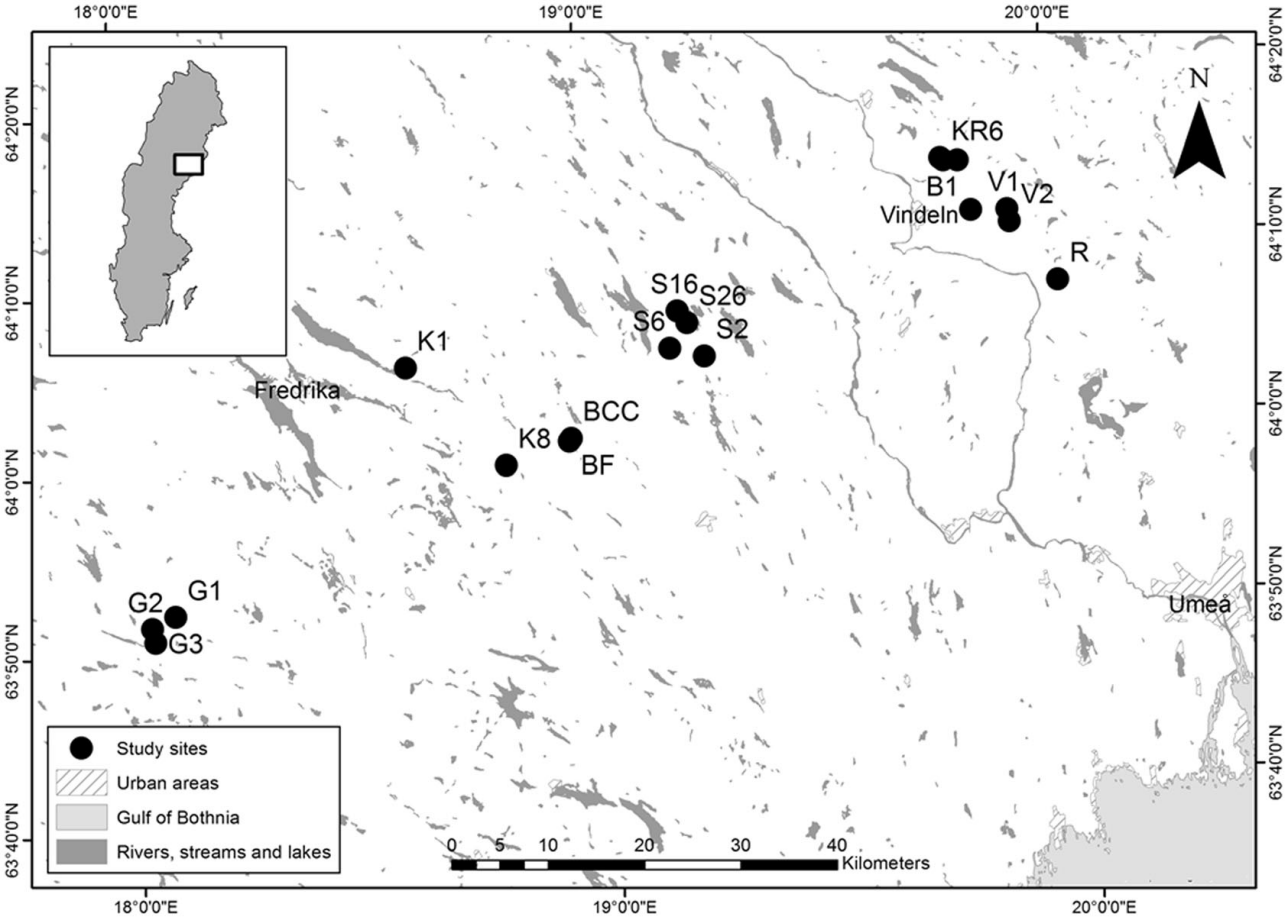
Locations of study sites in northern Sweden, including map coordinates. The *inset* shows the location of the study region in Sweden

The 18 study sites and their catchments (Table 1) are all situated in the boreal forest of northern Sweden (Fig. 1) and were selected to represent a land-use gradient while being similar in slope, width, and depth. For these 1st to 2nd order streams, elevation above sea level (m a.s.l.), catchment size (ha), land cover (percentage of forest, mire, and lake), and proportions of different forest regeneration were determined from 25 × 25 m digital elevation models using the Watershed tool within the Spatial Analyst toolbox in ArcMap version 10. For this, two map sources were used; Swedish Topographic Map (Terrängkartan; 1:50 000) and Forest Map (Skogskarta; 1:50 000). All 18 catchments were dominated by forest and did not contain agricultural land use. Forest regeneration classes were organized according to years following clear-cutting: 0–10, 11–50, 51–100, and 101–300, which represent deciduous-dominated, mixed, coniferous-dominated, and old-growth stands, respectively.

**Table 1:** Geographical, land-cover, and land-use characteristics of the study sites and their catchments

Site	Latitude	Longitude	Elevation (m a.s.l.)	Catchment size (ha)	Land cover (%)			Forest regeneration age class (%)			
					Forest	Mire	Lake	0–10	11–50	51–100	101–300
B1	64°12′06	19°49′43	215	181.9	78.1	21.9	0	12.5	17.3	54.7	9.1
B3	64°00′43	18°56′32	279	156.0	97.4	2.6	0	2.4	42.8	45.6	7.4
B4	64°00′52	18°56′50	271	41.0	93.2	6.8	0	57.4	9.3	21.9	9.4
G1	63°52′06	18°05′23	302	112.0	79.6	20.4	0	5.4	28.6	47.7	10.9
G2	63°51′29	18°02′25	404	109.0	88.6	7.3	4.0	4.0	54.2	25.0	4.1
G3	63°50′43	18°02′46	415	50.0	95.6	4.4	0	12.4	22.5	48.9	12.9
KB1	64°05′20	18°36′15	362	82.8	87.1	8.9	0	2.7	26.6	49.2	8.6
KB8	63°59′35	18°48′22	241	64.0	79.1	20.9	0	1.2	54.6	18.8	6.3
KR1	64°14′55	19°48′28	223	45.0	97.9	2.1	0	0.6	3.9	50.3	25.6
KR6	64°15′07	19°46′16	237	100.0	69.7	27.0	3.3	0	0.4	30.5	52.8
KR7	64°14′59	19°46′39	232	47.0	82.1	17.9	0	3.0	13.4	28.9	54.6
R1	64°07′51	20°00′08	172	392.0	88.3	11.2	0.4	10.4	24.2	49.4	7.3
S2	64°04′59	19°14′24	250	37.0	69.8	30.2	0	0	11.2	60.7	9.5
S6	64°05′33	19°10′06	254	89.0	96.2	3.8	0	10.2	30.6	49.0	7.0
S16	64°07′36	19°11′20	222	593.8	59.1	40.5	0.2	10.4	20.2	37.3	13.0
S26	64°06′54	19°12′28	222	18.0	100.0	0	0	0	36.0	42.4	21.6
V1	64°12′00	19°54′20	188	167.8	92.2	7.8	0	9.4	26.3	53.7	8.1
V2	64°11′18	19°54′32	203	253.5	81.2	18.8	0	5.4	30.6	50.4	6.1
${\bar{\text{x}}}$			261	141.1	85.3	14.0	0.4	8.2	25.1	42.5	15.2

### Data collection

In late September 2012, study sites at each of the 18 streams were selected as a 50-m reach containing riffles. At both ends and in the middle of each study reach, a spherical densiometer was used to measure canopy openness. At the upstream end of each reach, we measured water temperature and took water samples for analysis of pH, dissolved organic carbon (DOC), dissolved inorganic nitrogen (DIN), and soluble reactive phosphorus (SRP). Water samples for DOC, DIN, and SRP were filtered on site (0.45-µm nylon membrane filters, Sarstedt, Nümbrecht, Germany). All samples were kept cold during the day and later stored in a refrigerator (pH and DOC) or frozen (–20°C; DIN and SRP) for analysis within a few days or weeks, respectively. DOC and total dissolved nitrogen (TDN) were analysed by a Shimadzu TOC-V_CPH_ analyzer (Shimadzu, Duisburg, Germany). NO_3_^−^ (Method G-384-08 Rev. 2), NH_4_^+^ (Method G-171-96 Rev. 12), and SRP (Method G–297-03 Rev. 1) were analysed using a SEAL Analytical AutoAnalyzer 3 (SEAL Analytical, Wisconsin, USA).

In late September, we used a Surber sampler with a basal area of 20 × 25 cm (0.05 m^2^) to collect stream macroinvertebrates. At each site, five samples were taken at randomly selected locations. Stream depth and water velocity (Electromagnetic Open Channel Flow Meter, Model 801; Valeport, Totnes, UK) were also measured at each sampling location. The samples were obtained by disturbing the substrate within the Surber sampler by hand for 60 s. Gravel and fine inorganic and organic streambed materials were collected in the Surber net. Cobbles were transferred to a water-filled bucket and scrubbed separately to collect animals attached to those surfaces. All the collected material from each sample was placed in a separate Whirl–Pak^®^, along with 10 ml of 96 % ethanol. Samples were stored at 6 °C before being sorted.

In the laboratory, samples were separated into macroinvertebrates and coarse-particulate organic matter (CPOM). The CPOM was further divided into deciduous leaf litter, coniferous needle litter, cones and twigs (hereafter, ‘small woody debris’ [SWD], i.e. <2 cm in diameter), and aquatic moss for estimates of litter standing stock of different qualities and aquatic moss abundance at each site. Each class of CPOM was dried (60 °C) to a constant biomass, weighed, ashed (550 °C for 40 min), and then re-weighed to obtain the ash-free dry mass (AFDM). The macroinvertebrates were preserved in 70 % ethanol, before being sent to a certified taxonomist for determination.

In total, 73 taxa were identified and these were used to calculate total taxonomic richness and diversity (Shannon Wiener index, H′), community composition using principal component analysis (PCA) on absolute (pooled) abundances, and the proportional abundance of Simuliidae and Chironomidae, as these two taxonomic groups were among the most abundant. For all community measures, the five subsamples at each site were pooled, to obtain measures at the site level. Further, we assigned functional traits to the macroinvertebrate taxa (Poff et al. 2006) using an extensive European freshwater database (Schmidt-Kloiber and Hering 2012). Functional traits were not assigned to taxa not identified to a high enough resolution (e.g. Nematoda). This process rendered 21 functional traits, each with two to five modalities, for 41 macroinvertebrate taxa (Supplementary Tables S1 , S2 ). These data were used to calculate functional trait diversity (Shannon Wiener index, H′) and the proportion of individuals with low pH sensitivity.

In July 2013, we characterized the benthic substrate composition at each stream. For this, the intermediate axis of 200 gravel/cobbles was measured using random walk sampling. The mineral substrate was classified into different size categories with particles <2 mm (i.e. sand) as the smallest category. In cases where only fine organic particles were found at the random location, particles were classified as zero (and later as ‘organic fines’). Data on the mineral substrate size classes were used to calculate median substrate size and substrate heterogeneity (i.e. Shannon Wiener index, H′).

### Statistical methods

We used partial least squares (PLS) regression to explore relationships between different invertebrate metrics, in-stream habitat variables, and catchment attributes. More specifically, we analysed how catchment-scale land use (i.e. forest regeneration age classes) and land cover explained variation in both in-stream physico-chemical conditions and headwater macroinvertebrate metrics. Further, to assess the relative importance of catchment-scale descriptors versus in-stream variables for headwater invertebrate metrics, we performed separate analyses with in-stream variables as the only predictor variables. PLS relates two data matrices (including predictor and dependent variables) to each other by a linear multivariate model and produces latent variables (PLS components) extracted from predictor variables that maximise the explained variance in the dependent variables. PLS is especially useful when predictor variables are correlated and when the number of predictor variables is high (Carrascal et al. 2009). The evaluation of the PLS models was based on the level of variance explained (*R* ^2^), loadings of the independent variables, and the variable influence on projection (VIP). The independent variable loading describes the relative strength and direction of the relationship between independent and response variables. The VIP value summarises the importance of each variable, and, as a limit for when a predictor variable is important in a model, we chose VIP > 1.0.

To visualize relationships between macroinvertebrate taxa and environmental conditions, canonical correspondence analysis (CCA) was performed (and plotted), using the predictor variables that were the most important (i.e. VIP > 1.0) in the PLS models for macroinvertebrate community PC1 and PC2. Dependent variables were ln transformed, if necessary, to meet the assumptions of normality and equal variance, and assumptions were checked using standard diagnostics. PLS regression analyses were performed using XLSAT (XLSTAT 2015.2.01, Addinsoft SRAL, Germany), and CCA were performed using the vegan library (Oksanen et al. 2014) in R (R Core Team 2012).

## Results

Among sites, elevation varied by a factor of 2.4, while catchment size varied by a factor of 33 (Table 1). The proportion of mire in catchments ranged from 0 to 40.5 % and was not significantly related to any of the forest-age categories. Lakes were absent in most catchments and were therefore not included in the statistical analyses. Hence, all catchments were dominated by forest, and in these forests, stands of 11–50 and 51–100 years in age were the most common (Table 1). At two sites (KR6 and KR7), mature forests (101–300 years) dominated, while recently clear-cut forest (0–5 years) was the most common regeneration class at one site (B4; Table 1).

Mean depth, water velocity, and water temperature were similar among sites (Table 2). Most canopies were relatively closed (<20 % openness), apart from B4, which was a recently clear-cut site, whose canopy was largely open (83.3 %). There was a positive relationship between proportion of young forest (0–10 years) in the catchment and reach-scale canopy openness (data not shown), and although this relationship was driven by one site (B4), it indicates that catchment-scale forest-age composition can be broadly reflected in reach-scale canopy openness. Sites varied from acidic to almost circumnetural (i.e. pH of 4.4–6.3) and concentrations of DOC and SRP varied from 9.8 to 42.4 mg C L^−1^ and 2.7 to 11.0 µg P L^−1^, respectively (Table 2). Importantly, pH, DOC, and SRP tended to co-vary among sites, such that sites with low pH tended to have both high DOC and SRP. Concentrations of DIN were generally less than 50 µg N L^−1^ with the exception of B4 (151.0 µg N L^−1^). The standing stock of organic matter (OM) was comprised mostly of SWD (54.4 ± 0.3 % [mean ± 1 SD]), while coniferous needle litter was the least abundant, and aquatic moss biomass varied substantially among sites (Table 2). There was some variation in median substrate size and substrate diversity among sites, but only two sites (G3 and KB8) showed a substantial cover (>50 %) by organic fines (Table 2).

**Table 2:** Stream physical characteristics, canopy openness, water chemistry, OM standing stock, bottom substrate characteristics, and macroinvertebrate taxonomic richness and abundance (the sum of all subsamples per site), for the study sites. Dissolved inorganic nitrogen (DIN) represents the sum of NO_3_-N and NH_4_-N. *SWD* small woody debris, *DOC* dissolved organic carbon, *SRP* soluble reactive phosphorus

Stream variables																	
Site	Mean depth (cm)	Mean velocity (m s^−1^)	Mean temp (°C)	Canopy open. (%)	Water chemistry				OM standing stock (g AFDM m^−2^)				Bottom substrate			Macroinvertebrates (0.25 m^−2^)	
					pH	DOC (mg L^−1^)	DIN (µg L^−1^)	SRP (µg L^−1^)	Leaf	Needle	SWD	Moss	Div. (H′)	Median size (cm)	Organic fines (%)	Richness	Abundance
B1	15.8	0.58	4.5	7.5	5.2	24.1	24.3	5.2	8.2	1.0	23.8	0	1.61	24.5	0	21	1689
B3	15.9	0.45	6.7	15.8	5.2	24.6	11.0	6.0	10.2	0.8	1.6	51.0	1.81	10	2.4	19	326
B4	8.9	0.36	6.9	83.3	4.7	34.8	151.0	11.0	3.2	0.4	2.2	0.2	1.82	34	0	15	2475
G1	19.3	0.55	4.5	19.2	4.7	17.0	13.5	5.9	5.2	0.4	4.2	39.8	1.90	33.5	0	17	693
G2	19.9	0.41	5.3	2.6	5.8	18.0	17.7	2.9	7.2	8.0	117.0	49.2	2.00	53	0	38	1921
G3	19.7	0.09	5.1	8.8	6.1	9.8	11.8	5.3	2.8	5.0	107.4	3.6	1.77	1	59.5	15	105
KB1	16.8	0.44	5.8	31.5	5.7	20.4	18.5	6.3	36.4	1.0	24.2	22.4	1.97	23.5	0	21	322
KB8	16.7	0.40	5.7	9.3	5.3	21.2	19.8	2.7	54.2	0.8	34.4	73.4	1.82	1	51.3	20	976
KR1	9.2	0.41	6.0	5.2	5.1	28.4	46.8	4.8	10.6	1.2	40.0	3.8	1.54	15	1	12	127
KR6	13.8	0.40	6.8	5.7	5.2	20.5	31.3	4.6	5.6	2.2	21.4	0.6	1.41	7	0.5	16	634
KR7	13.8	0.40	6.0	3.4	4.6	30.9	27.2	8.6	12.4	2.0	25.6	6.2	1.82	23	0	15	826
R1	21.5	0.79	3.9	18.2	5.4	22.5	26.4	3.1	6.8	0.4	6.0	0.6	1.42	30	0	30	1145
S2	9.6	0.36	7.1	5.3	4.4	42.4	24.2	9.5	6.6	0.8	57.8	4.4	1.33	7.5	0	13	2462
S6	11.0	0.33	7.0	6.5	5.5	31.3	29.1	8.5	8.6	0.2	4.6	11.2	1.97	49	0.5	19	519
S16	20.4	0.37	6.8	14.9	5.0	34.9	27.9	3.1	8.4	0.8	53.4	1.6	1.96	93	0.5	23	336
S26	8.5	0.48	6.5	4.2	6.3	18.7	13.0	2.9	4.8	0.6	37.4	0.4	1.52	8	0	19	551
V1	14.3	0.36	6.3	11.0	5.6	25.9	44.7	3.1	1.4	0.6	41.0	13.0	1.76	12	0	27	804
V2	19.0	0.77	3.8	8.8	5.7	16.8	15.1	3.5	36.6	0.8	78.2	1.2	1.87	40.5	0	32	890
${\bar{\text{x}}}$	15.2	0.44	5.8	14.5	5.3	24.6	30.4	5.4	12.7	1.5	37.8	15.7	1.74	10	6.4	20.7	932.2

The proportion of younger forest (i.e. 11–50 years) was the most important catchment-scale predictor for explaining variation in in-stream conditions (Table 3). Specifically, proportion of younger forest was negatively related to concentrations of DOC and SRP and positively related to pH. Further, streams in catchments dominated by younger forest had higher aquatic moss abundance and greater standing stock of SWD. Land-cover characteristics were also important for several in-stream variables, but catchment size was significantly associated with only physical characteristics (i.e. substrate, depth, and water velocity), while elevation and percent mire in the catchment were also related to water–chemical properties (Table 3).

**Table 3:** Results from partial least squares regression (PLS) analyses of catchment-scale characteristics as predictors of in-stream or macroinvertebrate variables. Numbers represent loadings (including direction of relationship) of predictor variables that obtained a VIP > 1.0 and cumulative amount of response variable variation explained by the first (C1) and second (C2) model component. *SWD* small woody debris, *DOC* dissolved organic carbon, *SRP* soluble reactive phosphorus, *AFDM* ash-free dry mass, *PC* principal component

Response variables	Catchment-scale variables								
	Forest regeneration age class (%)				Land cover (%)				
	1–10	11–50	51–100	101–300	Catchment size (ha)	Elevation (m a.s.l.)	Mire (%)	R^2^Y C1	*R* ^*2*^ *Y C2*
In-stream variables									
Median substrate size (cm)					0.867		0.455	0.45	0.48
Substrate diversity (H′)		0.575				0.519		0.46	0.51
DOC (mg L^−1^)		−0.565				−0.557	0.463	0.34	0.42
pH		0.559					−0.630	0.42	0.47
SRP (µg L^−1^)	0.582	−0.574	−0.521					0.52	0.57
Needles (g AFDM m^−2^)						0.840		0.500	0.60
SWD (g AFDM m^−2^)		0.403				0.803		0.26	0.31
Aquatic moss (g AFDM m^−2^)		0.751						0.66	0.71
Depth (cm)		0.405			0.752			0.68	0.78
Water velocity (cm s^−1^)					0.611	−0.677		0.39	0.45
Macroinvertebrate variables									
PC 1		0.649		−0.454	0.498			0.42	0.48
PC 2			−0.422		−0.545	0.646		0.57	0.61
Taxonomic richness		0.604		−0.461	0.641			0.63	0.69
Taxonomic diversity (H′)		0.428		−0.413	0.632			0.40	0.57
Trait diversity (H′)		0.503			0.541		−0.480	0.38	0.53
Simuliidae (%)					−0.456	−0.533	0.504	0.31	0.48
Chironomidae (%)						0.799	−0.447	0.33	0.41
Low pH sensitivity (%)		−0.399				0.547	0.314	0.37	0.45

Macroinvertebrate taxonomic richness based on pooled samples at each site varied from 12 to 38 taxa and among-site variation in total abundance was considerable (84 to 2475 individuals per 0.25 m^2^, i.e. the sum of all subsamples per site; Table 2). PC1 and PC2 explained 32 and 17 % of the variation in macroinvertebrate community composition, respectively. PC1 was positively related to abundances of a diverse assemblage of taxa (e.g. *Brachyptera risi*, *Baetis rhodani*, *Bardeniella freyi*, *Hydraena gracilis*) and primarily negatively related to the abundance of *Nemurella picteti*. PC2 was positively related to the abundance of *Plectrocnemia conspersa* and Limnephilidae and negatively related to primarily the abundance of *Silo pallipes* and *Jungiella longicornis*. As for the in-stream variables, proportion of younger forests was a strong predictor variable and positively related to all measures of macroinvertebrate diversity and PC1 and negatively related to the abundance of taxa with low pH sensitivity (Table 3). In contrast, the proportion of old forest (i.e. 101–300 years) was negatively related to both taxonomic richness and diversity and to PC1. Catchment area was positively associated with all measures of richness and diversity and negatively associated with relative abundance of simuliid larvae and PC2 (Table 3). In addition, mire cover was negatively associated with trait diversity and positively related to relative abundance of Simuliidae and taxa with low pH sensitivity.

Compared to the catchment-scale assessment (Table 3), the reach-scale predictor variables explained a greater amount of variation in most macroinvertebrate measures (Table 4). Overall, pH and SRP, followed by DOC, stream depth, and water velocity were the most important environmental variables for explaining different descriptors of the macroinvertebrate community. PC1, taxonomic richness, and taxonomic and trait diversity all shared pH (positive), SRP (negative), and depth (positive) as significant predictors. In addition, organic matter standing stock (positive) was significant for PC1 and taxonomic diversity, water velocity (positive) for taxonomic richness and trait diversity, DOC (negative) for taxonomic and trait diversity, and substrate size (positive) for taxonomic richness (Table 4). Proportional abundance of Simuliidae larvae was the highest in streams with homogeneous substrate (including low amounts of SWD), low depth, and low pH, while the highest proportional abundance of Chironomidae larvae was found in contrasting conditions. Lastly, the highest proportional abundance of taxa that tolerate low pH was found in streams with high DOC and SRP concentrations and low pH (Table 4).

**Table 4:** Results from partial least squares regression (PLS) analyses of in-stream environmental conditions as predictors of macroinvertebrate variables. Numbers represent loadings (including direction of relationship) of predictor variables that obtained a VIP > 1.0 and cumulative amount of response variable variation explained by the first (C1) and second (C2) model component. *SWD* small woody debris, *DOC* dissolved organic carbon, *SRP* soluble reactive phosphorus, *AFDM* ash-free dry mass, *PC* principal component

In-stream variables	Macroinvertebrate variables							
	PC 1	PC 2	Taxonomic richness	Taxonomic diversity	Trait diversity	Simuliidae (%)	Chironomidae (%)	Low pH sensitivity (%)
Median substrate size (cm)			0.309					
Substrate diversity (H′)						−0.296		
DOC (mg L^−1^)				−0.379	−0.427	0.387	−0.479	0.284
pH	0.317		0.337	0.424	0.463	−0.525	0.459	−0.438
SRP (µg L^−1^)	−0.396		−0.430	−0.473	−0.514	0.348		0.441
Needle (g AFDM m^−2^)	0.415	0.577					0.324	
SWD (g AFDM m^−2^)	0.370			0.285		−0.317	0.405	
Aquatic moss (g AFDM m^−2^)		0.329						
Depth (cm)	0.386		0.443	0.424	0.330	−0.307		
Water velocity (cm s^−1^)		−0.438	0.385		0.322		−0.314	−0.607
Canopy openness (%)		−0.368						
R^2^Y C1	0.56	0.56	0.61	0.57	0.58	0.48	0.56	0.49
R^2^Y C2	0.78	0.65	0.75	0.70	0.73	0.65	0.82	0.65

**Fig. 2:**
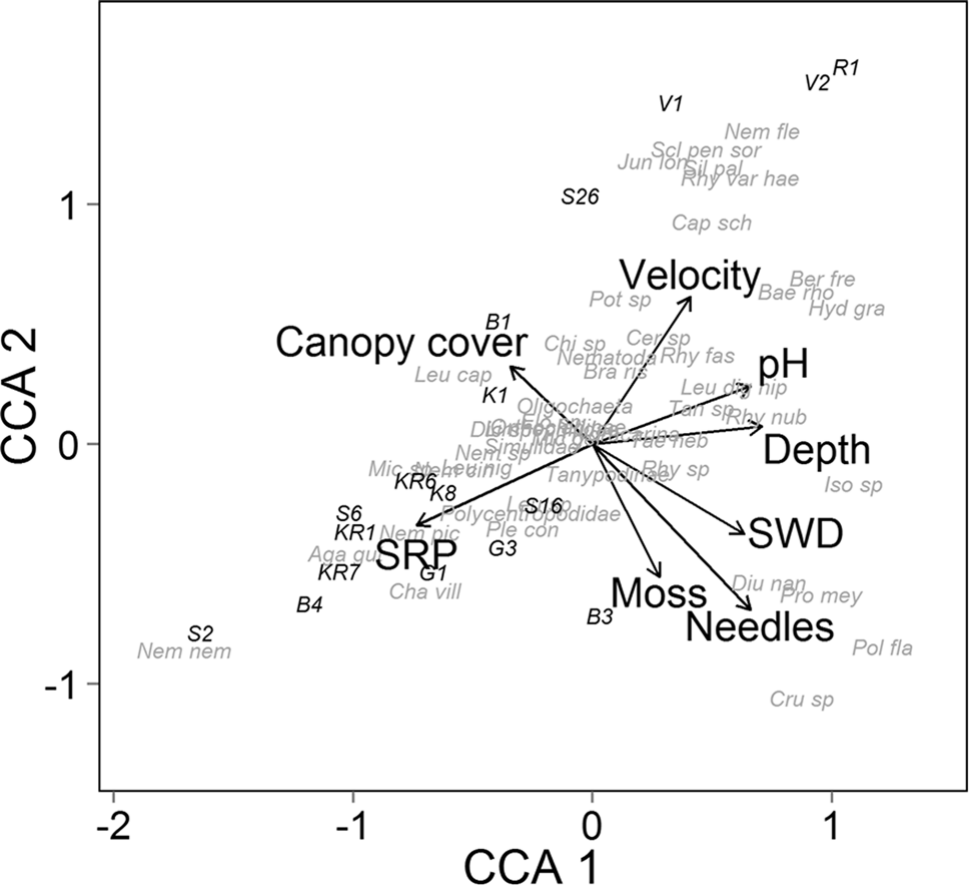
Two-dimensional output (primary and secondary axes) from a canonical correspondence analysis, using the main variables explaining stream macroinvertebrate community composition (represented in principal component [PC] axes PC1 and PC2), the macroinvertebrate taxa, and sites. Predictor variables are canopy cover (% openness), velocity (water velocity, m s^−1^), pH, depth (cm), small woody debris (SWD; g AFDM m^−2^), needles (g AFDM m^−2^), moss (aquatic moss, g AFDM m^−2^), and soluble reactive phosphorus (SRP; µg L^−1^). Length of the vector associated with predictor variable indicates the relative strength of each relationship. Abbreviated taxon names are the first three letters of the genus and species names. Where visible (from *top* to *bottom)*, Nem fle *Nemoura flexuosa*, Scl pen sor *Scleroprocta pentagonalis*/*sororcula*, Jun lon *Jungiella longicornis*, Sil pal *Silo pallipes*, Rhy var hea *Rhyapholophus varius*/*haemorrhoidalis*, Cap sch *Capnopsis schilleri*, Ber fre *Berdeniella freyi*, Bae rho *Baetis rhodani*, Pot sp *Potamophylax* sp., Hyd gra *Hydraena gracilis*, Cer sp *Ceratopogoninae*, Chi sp *Chironomini* sp., Rhy fas *Rhyacophila fasciata*, Bra ris *Brachyptera risi*, Leu cap *Leuctra capnoposis*, Leu dig hip *Leuctra digitata*/*hippopus*, Tan sp *Tanytarsini* sp., Rhy nub *Rhyacophila nubila*, Tae neb *Taenypoteryx nebulosa*, Nem sp *Nemoura* sp., Mic sp *Micropterna* sp., Rhy sp *Rhyacophila* sp., Iso sp *Isoperla* sp., Ple con *Plectrocnemia conspersa*, Nem pic *Nemurella picteti*, Aga gut *Agabus guttatus*, Diu nan *Diuera nanseni*, Cha vil *Chaetopteryx villosa*, Pro mey *Protonemura meyeri*, Nem nem *Nemoura*/*Nemurella* sp., Pol fla *Polycentropus flavomaculatus*, and Cru sp *Crunobia* sp

In the CCA, CCA1 and CCA2 explained 37 and 22 % of the variation, respectively. The two-dimensional CCA plot showed that CCA1 represented gradients in organic matter standing stock, pH, and depth, and, in the opposite direction, SRP, while CCA2 represented gradients in water velocity and canopy cover (% openness) in one direction and standing stock and moss abundance in the opposite direction (Fig. 2). While several of the 73 taxa fell in the middle of both axes (i.e. their distributions were not well explained), some taxa were strongly associated with the environmental gradients. Most notably, a diverse assemblage of diptera, stonefly, caddisfly, mayfly, and beetle taxa were positively associated with water velocity, and to some extent pH, and negatively associated with SRP, while several of taxa (and in particular Nemoura sp.) were found under contrasting environmental conditions (full list of taxa names provided in Fig. 2). Finally, several stonefly, caddisfly, and dipteran taxa were strongly associated with high organic matter standing stock and low canopy openness (Fig. 2).

## Discussion

**Fig. 3:**
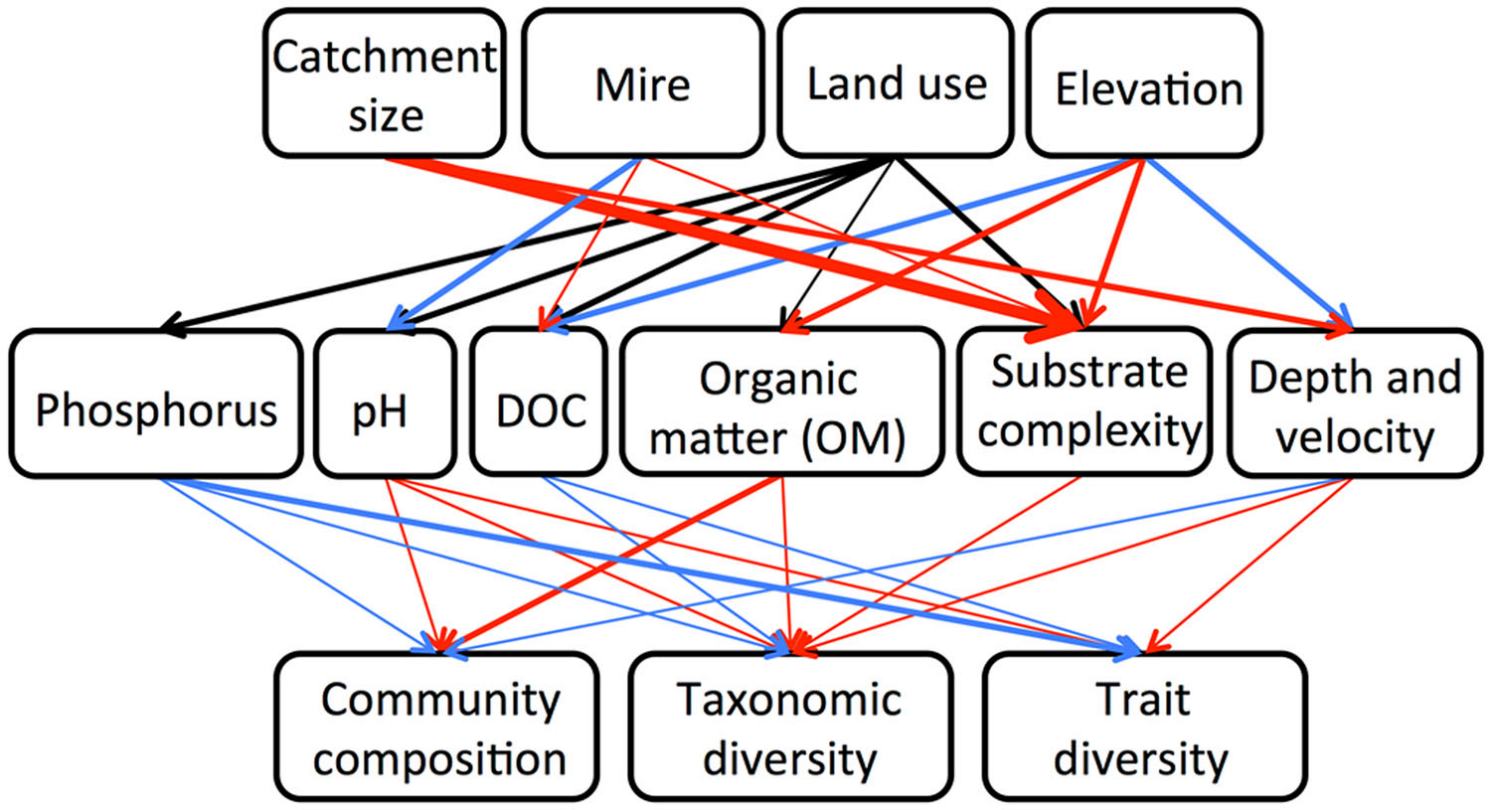
Summary of results from the separate partial least squares regression analyses on predictors of in-stream environmental conditions (Table 3) and predictors of macroinvertebrate variables (Table 4). *Arrow* thickness indicate loading size (i.e. level of importance), where a *thin line* represents < 0.50, medium thickness 0.5–0.79, and thick line >0.80. Red and blue indicate positive and negative relationships, respectively. As relationships between land use and response variables (*black*) differ among forest regeneration age classes, directions of relationships (see Tables 3, 4) are not given. Community composition entails macroinvertebrate PC1 and PC2 and %Simulidae and %Chironomidae, and both trait and taxonomic diversity are Shannon Wiener index (H′)

While headwaters are often touted as important habitats from a biodiversity perspective (e.g. Meyer et al. 2007), syntheses of published studies on small streams reveal a wide range in macroinvertebrate richness (e.g. 4–93 taxa; Clarke et al. 2008). The taxonomic richness observed across our sites (13–38 taxa) falls at that low end of this range, but is similar to other studies from boreal streams in Fennoscandia (e.g. Annala et al. 2014). Comparatively low richness in boreal headwaters may reflect natural constraints imposed by physical and chemical conditions that limit ecosystem productivity (e.g. Cardinale et al. 2009) and/or restrict individual species locally (e.g. through natural acidity; Petrin et al. 2007). Whether these community properties are additionally influenced by anthropogenic stressors is challenging to resolve in northern Sweden, where forest management has been sufficiently widespread that finding comparable, unaffected streams is a major obstacle. Nevertheless, results from this study indicate that variation in macroinvertebrate community composition across headwater streams in this region reflects a combination of natural drainage features and forest-management history within catchments (Fig. 3). Most macroinvertebrate community metrics were best explained by in-stream variables that, in turn, were often more strongly related to catchment land use rather than to natural drainage characteristics.

The strongest catchment-scale pattern was that increased drainage size corresponded to greater taxonomic richness and diversity of macroinvertebrate communities. This pattern is consistent with predictions of increasing diversity from low- to mid-order streams (Vannote et al. 1980), based on the idea that greater environmental heterogeneity (e.g. in light, temperature, resources) with channel size promotes a larger number of species (Minshall et al. 1985). Similar increases in stream macroinvertebrate richness/diversity across stream orders have been observed in Fennoscandia (e.g. Malmqvist and Hoffsten 2000; Heino et al. 2005); however, our results suggest that even within the range of low-order streams considered here, small increases in drainage area may be linked to richer communities. The mechanisms underlying this pattern between drainage area and community structure remain unresolved, although our results point to habitat heterogeneity (i.e. substrate properties, depth, and water velocity) as a potential link in these streams.

The relative cover by mires was also predictive of variation in some macroinvertebrate metrics across sites. Mires are dominant drainage features in northern Sweden, covering approximately 25 % of the landscape (Nilsson et al. 2001), and their outlet streams tend to have high concentrations of DOC and low pH, driven largely by organic acidity (Laudon et al. 2011b). Moreover, past studies in this region have shown strong positive correlations between DOC and SRP (Jansson et al. 2001), as well as elevated P concentrations in mire-outlet streams (Jansson et al. 2012). These three variables can thus co-vary in space, yet it is most likely the direct influence of acidity (rather than DOC or SRP) that is of most relevance to macroinvertebrate communities (Petrin et al. 2007). Correspondingly, in our study, streams with elevated mire cover showed lower trait diversity, higher abundance of acid-tolerant taxa, and were to a greater extent dominated by simuliid larvae. As such, our results, together with those from previous studies in the region (e.g. Petrin et al. 2007), suggest that low pH resulting from the presence of headwater mires represents a ‘filter’ (Poff 1997) on headwater macroinvertebrate communities, which are likely to be simplified both taxonomically and functionally. Such simplified communities may be relatively insensitive to additional stressors, such as those stemming from land use in the catchment (Annala et al. 2014).

Although our results show that part of the relationship between water chemistry (i.e. DOC, SRP, and pH) and assemblage structure is connected to mire cover, the distribution of forest stand ages in the catchment is also an underlying driver of these effects. Such relationships may be linked to recent clear-cuts, which are well known to cause a variety of changes in catchment properties that in turn influence stream chemistry (see review by Kreutzwiser et al. 2008). However, Palviainen et al. (2014) suggest a 30 % threshold in the cover of clear-cut forests necessary to see clear effects on water chemistry in boreal streams, and only one of our sites (B4) met this threshold. Indeed, at this site, multiple clear-cutting responses have been reported, including increased specific discharge (Sørensen et al. 2009), elevated DOC (Schelker et al. 2012) and DIN (Schelker et al. 2016) concentrations, and dramatically increased rates of microbial biofilm growth (Burrows et al. 2015). Not surprisingly, we observed invertebrate community responses that reflect these changes in basal productivity, including relatively high overall abundance, low taxonomic richness, and a notably high density (240 individual m^−2^) of large, cased caddisflies (*Chaetopteryx villosa*) that were rarely observed in the other streams. Similar community responses to clear-cutting have previously been described (e.g. Wallace and Gurtz 1986). However, this condition is likely a local and short-lived phenomenon in the boreal landscape, as elevated nutrient concentrations from clear-cuts do not appear to travel far downstream (Schelker et al. 2016) and only persist for 5–10 years following harvest (Futter et al. 2016). Increased inputs of fine sediments following clear-cutting could have longer-lasting impacts (Futter et al. 2016), but our sites are at or above the former highest coastline, where sediment supply from low gradient, geologically older landscapes is thought to be weak, even following disturbance (Rosenfeld et al. 2011).

In contrast to these previously observed short-term effects of clear-cutting, our results also indicate that increasing cover by younger (11–50 years) forest stands increased macroinvertebrate diversity, through influences on both water chemistry and benthic organic matter. Stand regeneration following clear-cutting is often characterized by a relatively greater proportion of productive deciduous trees (birch) that are gradually replaced by conifers as stand ages increase. Young- to middle-aged regenerating stands are productive, and may influence stream chemistry via higher demand for nutrients and water when compared to recently harvested and older stands. In addition, greater cover by deciduous trees may correspond to elevated pH (Finzi et al. 1988; Smolander and Kitunen 2002) and lower DOC concentrations (Cronan and Aiken 1985) in soil solution. Hence, assuming that the main driver of macroinvertebrate community composition is pH (from organic acidity), headwaters running through catchments dominated by younger, to a large extent deciduous, forests (11–50 years) should be more diverse in terms of taxa and traits (e.g. a wider range of pH sensitivity and functional feeding groups) than if clear-cuts or old, coniferous stands dominate the land cover. Our snapshot of stream chemistry and benthic communities supports this notion, yet mechanistic studies are needed to further explore the biogeochemical significance of birch forests in northern boreal soils and catchments.

The increasing levels of deciduous streamside vegetation in younger regenerating forests should, besides the influence of higher pH, support other factors that may promote headwater biodiversity. Deciduous or mixed forests are generally more open than coniferous (and especially spruce) forests (Naiman et al. 1987), allowing light to penetrate the canopy to stimulate in-stream primary production and produce higher-quality litter to in-stream detritivores than do conifers. However, in this study, standing stock of deciduous litter was not included in any predictive models and canopy openness was important only for PC2. There are several possible explanations for these somewhat unexpected results. First, given that deciduous litter is of high quality and therefore a rapidly diminishing resource in these headwaters, we might have failed to capture the true amounts received by our study sites. Second, macroinvertebrate communities in our study region might be well adapted to dark, nutrient poor conditions, with low levels of high-quality litter input. Instead, other environmental conditions, such as low pH, may simply be the more important species filter. Third, poor-quality litter, such as needles and SWD, can be important as it creates substrate and is a slowly diminishing resource that last throughout the long winters. Lastly, the abundance of aquatic moss was positively associated with cover of younger forests. It is well known that aquatic moss provides important habitats, and aquatic moss abundance can be influenced by stream pH (Tessler et al. 2014). Hence, young-forest cover and macroinvertebrate diversity may be linked via higher pH and subsequently greater moss abundance. Overall, a better understanding of the ecological significance of maintaining deciduous trees within catchments and riparian zones will aid in the management of these boreal landscapes.

Boreal landscapes comprise a mosaic of forest, lake, and mire patches that interact to shape spatial and temporal patterns in the physical and chemical characteristics of streams (Laudon et al. 2011b). Our results suggest that this template also constrains the regional variation in the structure of headwater communities. Despite the complexity of our results, pH and DOC (i.e. organic acidity) emerged as highly influential factors for macroinvertebrate community structure. Our results also suggest that forest land use, in addition to land cover (i.e. mires), impacts headwater biodiversity through its influence on water chemistry and OM loading. Our findings indicate that the highest macroinvertebrate diversity can be found in boreal catchments containing a high proportion of younger (11–50 years) regenerating forest, potentially due to a reduction in the production and transport of acidic organic compounds (with a low pH) to streams. At the same time, macroinvertebrate communities were less diverse in catchments containing a high proportion of mature forest. These results connect macroinvertebrate communities to the successional changes on land; however, because Sweden’s boreal forests are heavily managed, it is not clear whether these communities are on a trajectory towards those expected in more pristine or old-growth conditions.

## Electronic supplementary material

Below is the link to the electronic supplementary material.

Supplementary material 1 (PDF 29 kb)
