# Supplementary material for: Land use influences macroinvertebrate community composition in boreal headwaters through altered stream conditions
Source: Ambio. 2016 Nov 1;46(3):311–23. doi: 10.1007/s13280-016-0837-y (PMC5347524; doi:10.1007/s13280-016-0837-y)
Supplement: Supplementary file 1 — Supplementary material 1 (PDF 29 kb) [file 13280_2016_837_MOESM1_ESM.pdf]

Ambio

Electronic Supplementary Material
This supplementary material has not been peer reviewed.

Title: **LAND USE INFLUENCES MACROINVERTEBRATE COMMUNITY COMPOSITION IN BOREAL HEADWATERS THROUGH ALTERED STREAM CONDITIONS**

Micael Jonsson, Ryan M. Burrows, Johan Lidman, Emma Fältström, Hjalmar Laudon, Ryan A. Sponseller

Table S1 Macroinvertebrate functional traits and trait modalities

Macroinvertebrate functional trait	Modalities
Life cycle	Semivoltine
	Univoltine
	Bi-/multivoltine
Development	Fast seasonal
	Slow seasonal
	Non-seasonal
Adult emergence synchrony	Poor
	Well
Adult life span	Very short
	Short
	Long
Ability to exit	No
	Yes
Drought survival	No
	Yes
Female dispersal	Low
	High
Flying strength	Weak
	Strong
Occurrence in drift samples	No
	Common
	Abundant
Maximum crawling rate	Very low
	Low
	High
Swimming ability	No
	Weak
	Strong
Attachment	Unattached
	Some attachment
Armoring	No
	Poor
	Good
Body shape	Streamlined
	Not streamlined
Mode of respiration	Tegument
	Gills
	Plastron
Adult size	Small
	Medium
	Large
Rheophily	Depositional
	Depositional/Erosional
	Erosional
Thermal preference	Cold
	Cold/Warm
Habit	Burrow
	Sprawl
	Cling
	Swim
Trophic habit	Gatherer
	Filterer
	Herbivore
	Predator
	Shredder
pH sensitivity	Low
	Medium
	High

Table S2 List of stream macroinvertebrate taxa that were assigned functional traits

**Macroinvertebrate taxa**	
**Plecoptera**	**Trichoptera**
Diura nanseni	*Rhyacophila fasciata*
Isoperla sp.	*Rhyacophila nubila*
Brachyptera risi	*Plectrocnemia conspersa*
Taeniopteryx nebulosa	*Polycentropus flavomaculatus*
Amphinemura sp.	*Philopotamus montanus*
Protonemura meyeri	*Hydropsyche saxonica*
Nemoura cinerea	*Chaetopteryx villosa*
Nemoura flexuosa	*Potamophylax nigricornis*
Nemoura sp.	*Micropterna* sp.
Nemurella pictetii	*Silo pallipes*
Capnopsis schilleri	*Sericostoma personatum*
Leuctra nigra	*Micrasema gelidum*
Leuctra digitata/hippopus	
Leuctra sp.	**Diptera**
	Simuliidae
**Ephemeroptera**	*Tanypodinae*
Baetis rhodani	*Orthocladiinae*
Baetis niger	*Chironomini*
	Tanytarsini
**Coleoptera**	*Berdeniella freyi*
Agabus guttatus	*Jungiella longicornis*
Hydraena gracilis	*Dicranota* sp.
Elmis aenea	*Eloeophila* sp.
	Ceratopogoninae
